# Chronic *N*‐acetyl cysteine treatment does not improve respiratory system performance in the *mdx* mouse model of Duchenne muscular dystrophy

**DOI:** 10.1113/EP091862

**Published:** 2024-06-12

**Authors:** Michael N. Maxwell, Anthony L. Marullo, Esther Valverde‐Pérez, Aoife D. Slyne, Ben T. Murphy, Ken D. O'Halloran

**Affiliations:** ^1^ Department of Physiology University College Cork Cork Ireland; ^2^ Departamento de Bioquímica y Biología Molecular y Fisiología, Facultad de Medicina Universidad de Valladolid Valladolid Spain; ^3^ Unidad de Excelencia Instituto Biomedicina y Genética Molecular (IBGM) Universidad de Valladolid‐CSIC Valladolid Spain

**Keywords:** antioxidant, diaphragm, inspiratory pressure, muscular dystrophy, respiratory EMGs

## Abstract

Duchenne muscular dystrophy (DMD) is characterised by respiratory muscle injury, inflammation, fibrosis and weakness, ultimately culminating in respiratory failure. The dystrophin‐deficient mouse model of DMD (*mdx*) shows evidence of respiratory muscle remodelling and dysfunction contributing to impaired respiratory system performance. The antioxidant *N*‐acetylcysteine (NAC) has been shown to exert anti‐inflammatory and anti‐fibrotic effects leading to improved respiratory muscle performance in a range of animal models of muscle dysfunction, including *mdx* mice, following short‐term administration (2 weeks). We sought to build on previous work by exploring the effects of chronic NAC administration (3 months) on respiratory system performance in *mdx* mice. One‐month‐old male *mdx* mice were randomised to receive normal drinking water (*n* = 30) or 1% NAC in the drinking water (*n* = 30) for 3 months. At 4 months of age, we assessed breathing in conscious mice by plethysmography followed by *ex vivo* assessment of diaphragm force‐generating capacity. Additionally, diaphragm histology was performed. In separate studies, in anaesthetised mice, respiratory electromyogram (EMG) activity and inspiratory pressure across a range of behaviours were determined, including assessment of peak inspiratory pressure‐generating capacity. NAC treatment did not affect force‐generating capacity of the *mdx* diaphragm. Collagen content and immune cell infiltration were unchanged in *mdx* + NAC compared with *mdx* diaphragms. Additionally, there was no significant effect of NAC on breathing, ventilatory responsiveness, inspiratory EMG activity or inspiratory pressure across the range of behaviours from basal conditions to peak system performance. We conclude that chronic NAC treatment has no apparent beneficial effects on respiratory system performance in the *mdx* mouse model of DMD suggesting limited potential of NAC treatment alone for human DMD.

## Abstract (highlights)

- **What is the central question of this study?**
  Does chronic treatment with the antioxidant, anti‐inflammatory and anti‐fibrotic dietary supplement *N*‐acetyl cysteine (NAC) improve respiratory system performance in the *mdx* mouse model of Duchenne muscular dystrophy?
- **What is the main finding and its importance?**
  Chronic treatment with NAC does not improve a range of respiratory‐related parameters in *mdx* mice suggesting limited potential for NAC alone as an intervention in Duchenne muscular dystrophy.

## 1 INTRODUCTION

Duchenne muscular dystrophy (DMD) is a fatal neuromuscular disease caused by mutations in the *DMD* gene, which results in the absence of the muscle isoform of the protein dystrophin (Dp427m) (Aartsma‐Rus et al., 2006; Hoffman et al., 1987). Dystrophin is part of the dystrophin glycoprotein complex (DGC), a structure that connects the myocellular cytoskeleton to the extracellular matrix via the sarcolemma, providing support during recurring contractions, and thus protecting muscle fibres (Gao & McNally, 2015). In the absence of dystrophin, during contraction, damage occurs to muscle fibres leading to muscle deterioration and degeneration, which is a primary phenotype of DMD (Burns et al., 2018, Burns, Drummond et al., 2019; Burns, Murphy et al., 2019; O'Halloran et al., 2023). As a result, the DGC and its function is lost and inflammation and fibrosis ensue with attendant respiratory muscle weakness, which is evident in reductions in ventilatory capacity, and eventual respiratory failure and death (De Bruin et al., 1997; Evans et al., 2009; Khirani et al., 2014; Smith et al., 1989). To date, there is no cure for people with DMD. Therapies for the improvement of respiratory muscle strength in DMD are necessary, to delay or prevent respiratory muscle failure, protecting respiratory system capacity.

The dystrophin‐deficient mouse model of DMD (*mdx*) exhibits impaired respiratory muscle performance like that observed in human DMD. Studies of the respiratory system of the *mdx* mouse have shown severe muscle weakness in conjunction with structural remodelling of the diaphragm (Burns et al., 2018, Burns, Drummond et al., 2019; Burns, Murphy et al., 2019; Burns, Roy et al., 2017; Mhandire et al., 2022; O'Halloran et al., 2023). Markers of inflammation including immune cell infiltration, elevated cytokine concentrations, elevated levels of reactive oxygen species and collagen deposition are evident in the *mdx* diaphragm (Burns, Drummond et al., 2019; Choi et al., 2016; O'Halloran et al., 2023). It has also been established that electromyogram (EMG) activity in the principal muscles of breathing is reduced, most likely reflecting impaired neuromuscular transmission of central respiratory neural activation critical for respiratory performance (Burns, Murphy et al., 2019; O'Halloran et al., 2023; Personius & Sawyer, 2006).

Antioxidants have been proposed as a potential therapy for respiratory muscle dysfunction, and several studies have yielded promising results in animal models including animal models of muscular dystrophy (Burns, Ali et al., 2017; Burns et al., 2018; Garegnani et al., 2021; Lewis & O'Halloran, 2016; Lewis et al., 2015, 2016; O'Halloran & Lewis, 2017). *N*‐acetyl cysteine (NAC) is a dietary antioxidant, a precursor to glutathione, which is safe for use in humans. NAC exerts antioxidant, anti‐inflammatory and anti‐fibrotic effects leading to improved muscle performance in a range of animal models of muscular dystrophy (Burns, Drummond et al., 2019; Pinniger et al., 2017; Redwan et al., 2023; Terrill et al., 2012; Whitehead et al., 2008). Pinniger et al. (2017) showed an increase in *mdx* mouse grip strength and extensor digitorum longus (EDL) muscle force after 6 weeks of 2% NAC treatment. Furthermore, the authors showed evidence of a reduction in muscle inflammation and oxidative stress (Pinniger et al., 2017). Terrill et al. (2012) showed that 1% NAC treatment for 1 week prevented exercise‐induced myofibre necrosis and reduced blood creatine kinase activity after exercise. Whitehead et al. (2008) showed that 6 weeks of 1% NAC treatment resulted in a reduction in reactive oxygen species by reduced fluorescence in muscle tissue stained for dihydroethidium, and a reduction of damage in the *mdx* EDL evidenced by a decrease in centrally nucleated muscle fibres. Redwan et al. (2023) reported that 2% NAC administration for 6 weeks significantly reduced EDL muscle mass and abnormal fibre branching and splitting, which causes dystrophic EDL muscle hypertrophy (Redwan et al., 2023). Previously our research group investigated the effects of acute NAC treatment on respiratory performance in the *mdx* mouse. Burns, Drummond et al. (2019) showed that 1% NAC treatment for 2 weeks rescued *mdx* diaphragm function by increasing force‐generating capacity, associated with a decrease in diaphragm fibrosis and immune cell infiltration compared to untreated *mdx* mice. However, short‐term NAC treatment did not recover impaired respiratory EMG activity in diaphragm and external intercostal muscles of breathing. Collectively, these studies suggest that NAC administration may be a promising therapeutic for the treatment of skeletal muscle dysfunction in the *mdx* model of DMD with potential application to human DMD.

DMD is a progressive muscle wasting disorder with temporal decline in respiratory system performance. It is important to consider the efficacy of longer‐term pharmacotherapies for DMD over time domains that reflect progressive deterioration in muscle performance due to persistent dystropathology. In the current study, we sought to assess the effects of chronic NAC treatment (1% NAC for 3 months) on respiratory system performance in *mdx* mice starting at 1 month of age. We hypothesised that NAC would have beneficial effects on dystrophic respiratory muscle structure and function and would improve respiratory EMG activity leading to preservation of respiratory performance.

## 2 METHODS

### 2.1 Ethical approval

Procedures on live animals were performed under project authorisation (AE19130/P117) from the Health Products Regulatory Authority in accordance with Irish and European law with prior ethical approval by University College Cork (AEEC 2019/013). Experiments were carried out in accordance with guidelines and requirements laid down by University College Cork's Animal Welfare Body.

### 2.2 Experimental animals and NAC treatment

Breeding pairs (homozygous females with hemizygous males) for *mdx* mice (C57BL/10ScSn‐Dmd*mdx*/J) were purchased from The Jackson Laboratory (Bar Harbor, ME, USA) and a colony was established at University College Cork's specific pathogen‐free facility. Animals were housed in individually ventilated cages in temperature‐ and humidity‐controlled rooms, operating on a 12 h light:12 h dark cycle with food and water available ad libitum. Studies were performed in 60 male *mdx* mice. Mice were randomly assigned to two different experimental groups: *mdx* (*n* = 30) and *mdx* + NAC (*n* = 30). The *mdx* + NAC group received 1% NAC (Sigma‐Aldrich, Wicklow, Ireland) in the drinking water for 3 months, beginning at 1 month of age. Drinking water containing NAC was pH matched to control water and prepared fresh each day. For the *mdx* + NAC group, water bottles were weighed daily. Estimated fluid intake was normal and stable over the 3‐month intervention, averaging 4–5 mL per mouse per day. Mice were studied at 4 months of age. A thorough assessment of respiratory performance was made, with measurements of breathing and ventilatory capacity in response to chemoactivation, recordings of thoracic inspiratory pressure and respiratory muscle electromyography (EMG) in anaesthetised animals and respiratory muscle function tests *ex vivo*. Diaphragm muscle tissue was collected for structural and molecular analysis using standard histological techniques and qPCR.

### 2.3 Plethysmography

Whole‐body plethysmography was used to assess respiratory flow in unrestrained, unanaesthetised *mdx* (*n* = 15) and *mdx* + NAC (*n* = 15) mice. Mice were introduced into plethysmograph chambers (Model PLY4211; volume 600 mL, Buxco Research Systems, Wilmington, NC, USA) and were allowed an acclimation period (∼2 h) with room air passing through each chamber (0.85 L min^−1^).

#### 2.3.1 Experimental protocol

Following acclimation, during confirmed periods of quiet rest, a 10‐min baseline recording was performed in normoxia. This was followed by combined central and peripheral chemoreceptor stimulation with hypercapnic hypoxia (10% O_2_ and 6% CO_2_) for 10 min, to examine maximum chemoactivated breathing. Respiratory parameters including respiratory frequency (*f*), tidal volume (*V*_T_) and minute ventilation (${\dot{V}}_{I}$) were recorded on a breath‐by‐breath basis for analysis offline. A gas analyser (ADInstruments, Colorado Springs, CO, USA) was used to measure oxygen consumption (${\dot{V}}_{O_{2}}$) and carbon dioxide production (${\dot{V}}_{CO_{2}}$).

#### 2.3.2 Data analysis

Baseline normoxic ventilation was determined as an average of the baseline period. For hypercapnic hypoxia, ventilatory measurements were taken during the final 5 min of the challenge to ensure steady‐state changes. *V*_T_ and ${\dot{V}}_{I}$ were normalised for body mass (g).

### 2.4 *Ex vivo* diaphragm muscle function

Following plethysmography measurements, mice were killed by cervical dislocation under 5% isoflurane anaesthesia, and diaphragm muscle (rib and central tendon intact) was immediately excised and placed in a tissue bath at room temperature containing continuously gassed hyperoxic (95% O_2_/5% CO_2_) Krebs solution (in mM: NaCl, 120; KCl, 5; calcium gluconate, 2.5; MgSO_4_, 1.2; NaH_2_PO_4_, 1.2; NaHCO_3_, 25; and glucose, 11.5) and d‐tubocurarine (25 μM) prior to functional analysis. Thin strips of diaphragm muscle were prepared from the mid‐costal section of the right hemidiaphragm. Diaphragm muscle preparations were suspended vertically between two platinum plate electrodes in a water‐jacketed tissue bath at 35°C containing Krebs solution and were continuously gassed with carbogen (95% O_2_ and 5% CO_2_). The rib was sutured to an immobile hook and remained in a fixed position for the duration of the experiment. Using non‐elastic string, the central tendon was attached to a lever connected to a dual‐mode force transducer (Aurora Scientific Inc., Aurora, ON, Canada). To determine muscle optimum length (*L*_o_), the length of the muscle preparations was adjusted using a micro‐positioner between intermittent twitch contractions. The muscle length which revealed maximal isometric twitch force for a single isometric twitch stimulation (supramaximal stimulation, 1 ms duration) was considered *L*_o_. Diaphragm preparations were maintained at *L*_o_ for the duration of the protocol.

#### 2.4.1 Experimental protocol

First, a single isometric twitch contraction was measured and tetanic force at 100 Hz (*P*_o_), time to peak (TTP) and half‐relaxation time were assessed. To examine the force–frequency relationship, muscle bundles were stimulated sequentially at 25, 50, 75 and 150 Hz (300 ms train duration). Contractions were interspersed by a 1 min interval.

#### 2.4.2 Data analysis

Muscle bundle cross‐sectional area (CSA) was determined for the purpose of normalising muscle force to bundle size. CSA was calculated by dividing muscle mass (weight in grams) by the product of muscle *L*_o_ (cm) and muscle density (assumed to be 1.06 g cm^−3^). Muscle force was divided by bundle CSA and expressed as specific force (N cm^−2^). TTP and half‐relaxation time were measured as indices of isometric twitch kinetics and were expressed in milliseconds.

### 2.5 Respiratory EMGs and inspiratory pressure recordings

In separate studies, *mdx* (*n* = 15) and *mdx* + NAC mice (*n* = 15), anaesthesia was induced with 5% isoflurane in 60% O_2_ (balance N_2_) in an induction chamber. Mice were subsequently placed in the supine position and received 2% isoflurane in 60% O_2_ (balance N_2_) by nose‐cone delivery. Mice were gradually transitioned from isoflurane to urethane anaesthesia (1.7 g kg^−1^ i.p. in total given in three injections) over a 25‐min period. Body temperature was maintained at 37°C via a rectal probe and thermostatically controlled heating blanket (Harvard Apparatus, Holliston, MA, USA). We established an acceptable surgical plane of anaesthesia, determined by an absent pedal withdrawal reflex and no somatic motor response to noxious pinch. Supplemental anaesthetic was administered as required. A pulse oximeter clip (MouseOx™, Starr Life Sciences Corp., Oakmount, PA, USA) was placed on a shaved thigh for the measurement of peripheral capillary O_2_ saturation ($S_{pO_{2}}$). A mid‐cervical tracheotomy was performed. All animals were maintained with a bias flow of supplemental O_2_ ($F_{iO_{2}}$ = 0.60) under baseline conditions. Oesophageal pressure was measured using a pressure‐tip catheter (Mikro‐Tip, Millar Inc., Houston, TX, USA), which was positioned in the thoracic oesophagus through the mouth. The catheter was advanced into the stomach to record positive pressure swings during inspiration and then withdrawn into the lower oesophagus where stable phasic sub‐atmospheric pressure swings during inspiration were observed. Concentric needle monopolar recording electrodes (26G; Natus Manufacturing Ltd, Gort, Ireland) were inserted into the middle costal region of the diaphragm on the right‐hand side for the continuous measurement of diaphragm EMG activity. In addition, concentric needle monopolar electrodes were inserted into an external intercostal (EIC), in the second to fourth rostro‐ventral intercostal space, for the measurement of EIC EMG and into the parasternal intercostal (PS) (second or third space) for the measurement of PS EMG. In addition, concentric needle monopolar electrodes were used to record scalene (SCAL), cleidomastoid (CM), sternomastoid (SM), sternohyoid (SH) (all mid‐belly insertions) and trapezius (TRAP) (superficial insertion in the upper region on either side) with contemporaneous recordings of eight EMG signals in each mouse. EMG signals were amplified (×5000), filtered (350 Hz low cut‐off to 5000 Hz high cut‐off) and integrated (50 ms time constant; Neurolog system, Digitimer Ltd, Welwyn Garden City, UK). All signals were passed through an analog‐to‐digital converter (Powerlab r8/30; ADInstruments) and were acquired using LabChart 8 (ADInstruments) sampled at 20 kHz (EMG) and 1 kHz for other parameters (tracheal airflow and pressure, and $S_{pO_{2}}$).

#### 2.5.1 Experimental protocol

Following instrumentation, animals were allowed to stabilise before baseline parameters were measured. Next, animals were challenged with a single sustained tracheal occlusion until peak inspiratory efforts (identified as stable, maximal successive efforts often until task failure) were observed in the inspiratory pressure recordings during sustained maximum non‐ventilatory efforts. Following recovery, animals were instrumented for the measurement of tracheal airflow, and parameters were recorded during a newly established second baseline (pre‐vagotomy) period. Subsequently, the vagi were sectioned bilaterally at the cervical level. Respiratory parameters were recorded under steady‐state conditions for a minimum of 10 min following vagotomy. Next, animals were challenged with hypercapnic hypoxia ($F_{iO_{2}}$ = 0.15/$F_{\mathrm{iC}O_{2}}$ = 0.06; 2 min) to examine the effects of chemostimulation on diaphragm, EIC, PS, CM, SM, SH, SCAL and TRAP EMG activity and ventilatory parameters. Following the experimental protocol, anaesthetised mice were killed by cervical dislocation and death was confirmed by the absence of cardiac rhythm.

#### 2.5.2 Data analysis

The amplitudes of absolute integrated inspiratory diaphragm, EIC, PS, CM, SM, SH, SCAL and TRAP EMG activity and peak inspiratory sub‐atmospheric oesophageal pressure change from baseline were measured and averaged under steady‐state basal conditions (typically over 1 min) and averaged for the five successive maximal sustained efforts (maximal response) of the single airway occlusion challenge (Burns, Murphy et al., 2019). During baseline breathing and chemoactivation, peak inspiratory and expiratory flows were measured, and inspiratory tidal volume was derived from the integral of tracheal airflow measurements.

### 2.6 Muscle histology

#### 2.6.1 Tissue preparation

Sections of hemidiaphragm from *mdx* (*n* = 8) and *mdx* + NAC mice (*n* = 8) were mounted on cubes of liver. Diaphragm samples were embedded in optimum cutting temperature embedding medium (OCT; VWR International, Dublin, Ireland) for cryoprotection and then frozen in isopentane (Sigma‐Aldrich) cooled on dry ice. Samples were then stored at −80°C for subsequent structural analysis. Serial transverse muscle sections (10 μm) were cut using a cryostat (Leica CM3050; Leica Biosystems, Nußloch, Germany) at −20°C and mounted across polylysine‐coated glass slides (VWR International) allowing for a distribution of tissue on a given slide.

#### 2.6.2 Histological analysis

To examine putative inflammatory cell infiltration of muscle, tissue sections were stained with haematoxylin and eosin (H&E) using an autostainer (Leica ST5010 Autostainer XL, Leica Biosystems). For percentage collagen deposition, picro‐sirius red (Leica Biosystems) staining was completed. Slides were mounted using DPX mounting medium (Sigma‐Aldrich), air‐dried and visualised on a bright field microscope (Olympus BX51, Hamburg, Germany) at ×10 magnification.

#### 2.6.3 Data analysis

A total of four tissue sections per animal were examined. Muscle histology was scored using ImageJ software. Putative inflammatory cell infiltration (the presence of cells in the extracellular matrix) was scored and expressed as a percentage of the total area of muscle. For slides stained with picro‐sirius red, the microscope lighting exposure was standardised during imaging. Images were analysed using a colour balance threshold and the area of collagen was expressed as a percentage of the total area of muscle. Data generated from multiple images with varying regions of interest per muscle were averaged per animal before computing group means.

### 2.7 Gene expression

#### 2.7.1 RNA extraction and quantification

Diaphragm muscle samples were stored at −80°C. Ten to twenty milligrams per sample was homogenised on ice in Tripure Isolation Reagent (Roche Diagnostics, Ltd, Burgess Hill, UK) using a bead homogeniser (Thermo Fisher Scientific, Waltham, MA, USA). Two chloroform‐phase separation steps were performed followed by 100% isopropanol precipitation of the RNA pellet and 70% ethanol wash. Isolated RNA was quantified by NeoDot (Neo Biotech, Nanterre, France). The integrity of isolated RNA was qualitatively assessed via gel electrophoresis (E‐gel, Thermo Fisher Scientific).

#### 2.7.2 Reverse transcription

Diaphragm muscle RNA was reverse transcribed into cDNA using a Transcriptor First Strand cDNA synthesis Kit (Roche Diagnostics Ltd) according to manufacturer's protocols.

#### 2.7.3 Quantification of gene expression by qPCR

cDNA was amplified using TaqMan Gene Expression Assays (FAM; Thermo Fisher Scientific) and Fast Start Essential Probe Master Mix (Roche Diagnostics) as per the manufacturer's instructions. All reactions were carried out on a 96‐well plate with a ratio of 5 μL (500 ng) cDNA/15 μL master mix using the Lightcycler 96 (Roche Diagnostics). Cycle threshold (*C*_T_) values were normalised to the reference gene, *Hprt1*. We have previously demonstrated that *Hprt1* is a suitable housekeeping reference gene in *mdx* diaphragm (Burns, Drummond, et al., 2019). The relative gene expression was calculated as ΔΔ*C*_T_ between normalised expression of the gene of interest to the reference gene (*Hprt1*). Changes in genes expression were calculated as a fold change relative to the control group (*mdx*).

### 2.8 Statistical analysis

Values are expressed as box and whisker plots (median, interquartile range (IQR) and individual data scatter plot) in graphs. Data were statistically compared by repeated measures two‐way ANOVA (or mixed model when occasional data points were missing for technical reasons) with Šidák's multiple comparisons *post hoc* test (Prism 10.2.0; GraphPad Software, Boston, MA, USA). Exact *P*‐values are reported for all comparisons. *P* < 0.05 was considered statistically significant.

## 3 RESULTS

### 3.1 Body mass and tibia length and mass measurements

**FIGURE 1:**
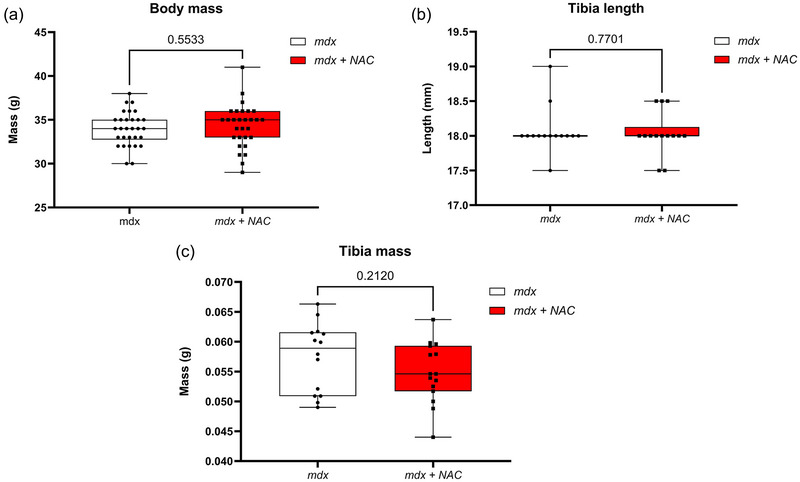
Body mass (a), tibia length (b) and tibia mass (c) in *mdx* and *mdx* + NAC mice. Values are expressed as box and whisker plots (median, 25−75 percentile and scatter plot) and were statistically compared using an unpaired two‐tailed *t*‐test. Exact *P*‐values are reported.

Figure 1 compares body mass and tibia length and mass between *mdx* and *mdx* + NAC groups. Body mass was unaffected by NAC administration. Tibia length and mass were also unaffected by NAC administration. Chronic NAC treatment had no effect on somatic growth and body mass in the *mdx* mouse model of DMD.

### 3.2 Baseline ventilation and ventilatory responsiveness to hypercapnic hypoxia in conscious mice

**FIGURE 2:**
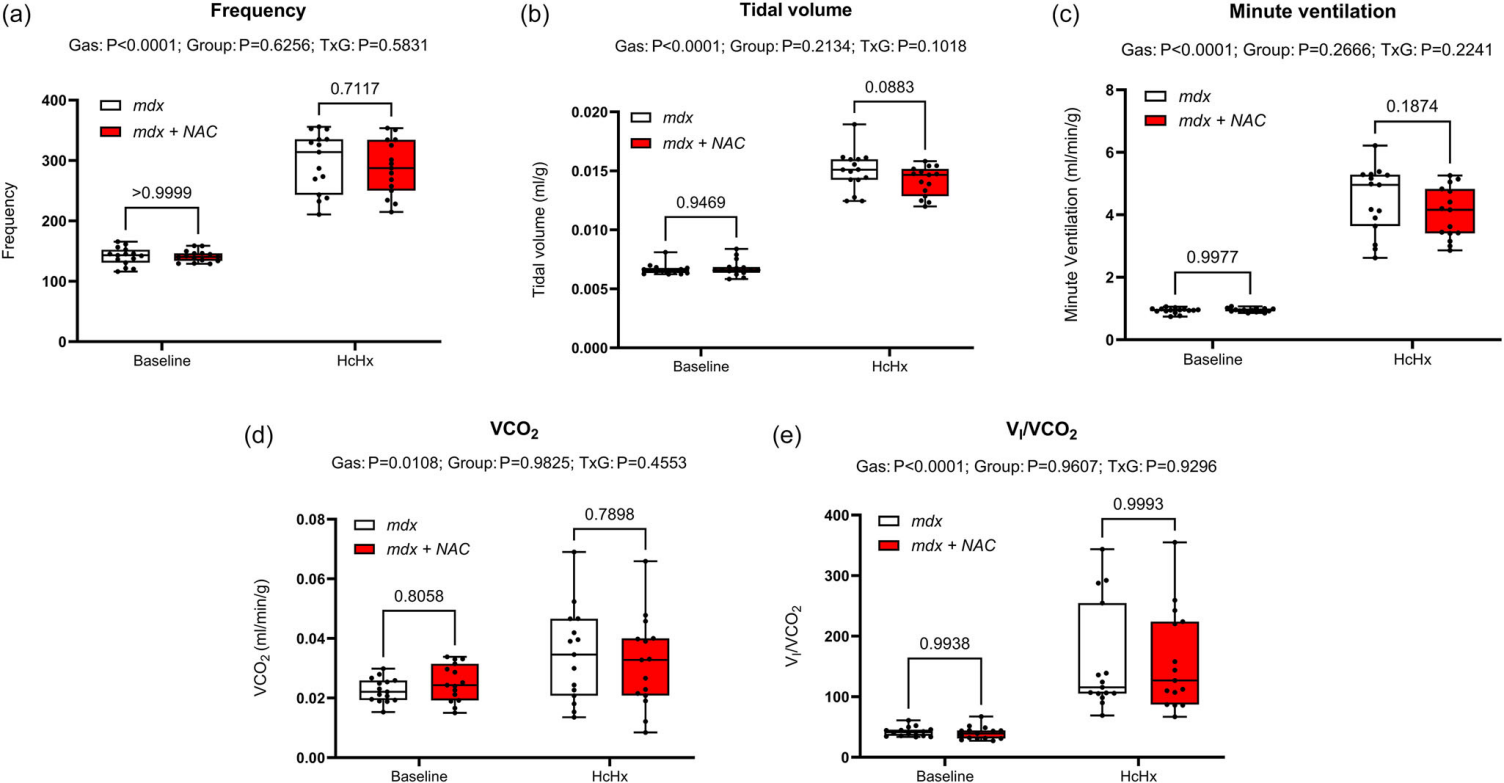
Assessment of breathing and metabolism during baseline and exposure to hypercapnic hypoxia (HcHx) in conscious *mdx* and *mdx* + NAC mice. Frequency (a), tidal volume (b) and minute ventilation (c) were determined from measurement of respiratory airflow. ${\dot{V}}_{CO_{2}}$ (d) was measured, and the ventilatory equivalent (${\dot{V}}_{I}$/${\dot{V}}_{CO_{2}}$) (e) was determined offline. Data were statistically compared by repeated measures two‐way ANOVA (or mixed model when occasional data points were missing for technical reasons) with Šidák's multiple comparisons *post hoc* test (Prism 10.2.0). Exact *P*‐values are reported for all comparisons.

Respiratory frequency, tidal volume (*V*_T_), minute ventilation (${\dot{V}}_{I}$), metabolic CO_2_ production (${\dot{V}}_{CO_{2}}$) and the ventilatory equivalent (${\dot{V}}_{I}$/${\dot{V}}_{CO_{2}}$) during baseline (20.9% O_2_) and hypercapnic hypoxia ($F_{iO_{2}}$ 0.10 and $F_{\mathrm{iC}O_{2}}$ 0.06) are shown in Figure 2. All parameters were equivalent between *mdx* and *mdx* + NAC mice. Chronic NAC treatment had no effect on ventilatory performance in the *mdx* mouse model of DMD.

### 3.3 Diaphragm muscle contractile function *ex vivo*

**FIGURE 3:**
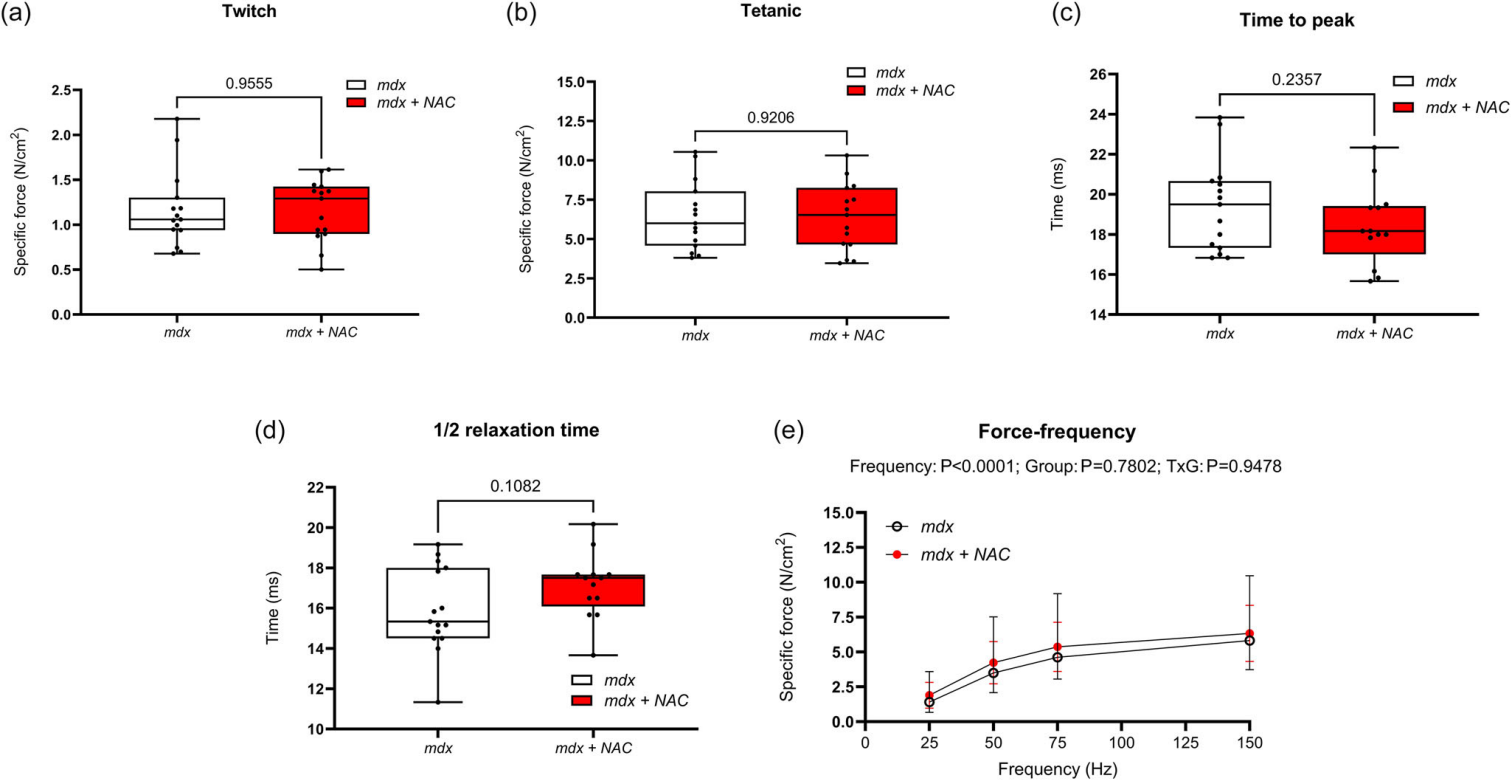
*Ex vivo* assessment of diaphragm twitch force (a), tetanic force (b), time to peak (TTP) (c), half‐relaxation time (d) and force–frequency relationship (e) in *mdx* and *mdx* + NAC mice. Values are expressed as box and whisker plots (median, 25−75 percentile and scatter plot) or mean ± SD (force–frequency plots). Force and contractile kinetics data were statistically compared using an unpaired two‐tailed *t*‐test. The force‐frequency relationships were compared by repeated measures two‐way ANOVA with Šidák's multiple comparisons post hoc test (Prism 10.2.0). Exact *P*‐values are reported for all comparisons.

Figure 3 shows summary data for diaphragm muscle twitch and tetanic contractions, time to peak, half‐relaxation time, and the force–frequency relationships in *mdx* and *mdx* + NAC mice. Twitch force, tetanic force, time to peak, half‐relaxation time, and the force–frequency relationship were equivalent between *mdx* and *mdx* + NAC mice. Chronic NAC treatment had no effect on diaphragm muscle function in the *mdx* mouse model of DMD.

### 3.4 Inspiratory pressure and obligatory and accessory muscle EMGs and ventilation in anaesthetised mice

**FIGURE 4:**
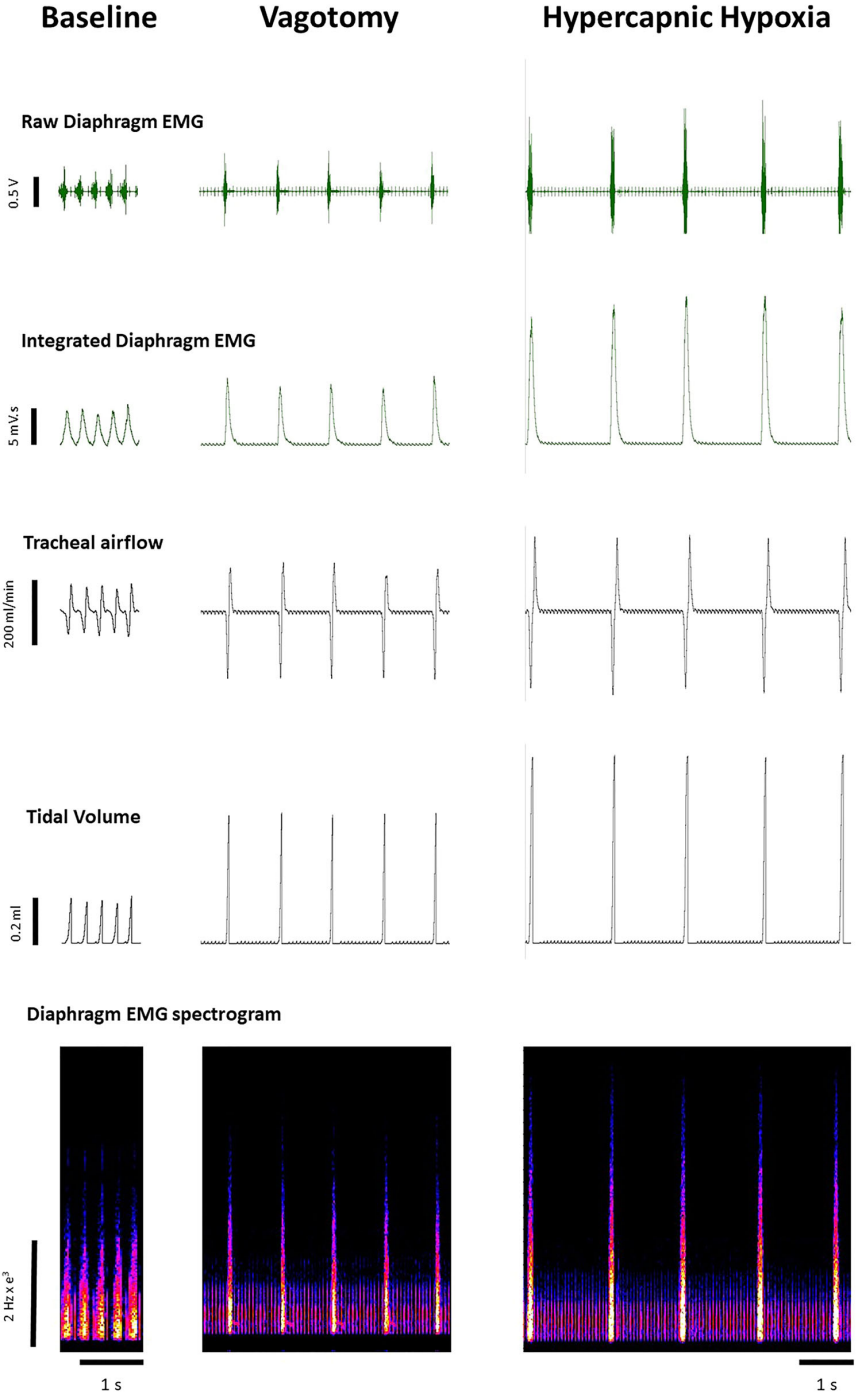
Original traces of respiratory parameters across the ventilatory range in an anaesthetised *mdx* mouse. Diaphragm electromyogram activity including raw, integrated and spectrogram traces, tracheal airflow and tidal volume in an anaesthetised *mdx* mouse during baseline, following vagotomy and during hypercapnic hypoxia.

Representative original traces of diaphragm electromyogram activity, tracheal airflow and tidal volume in an anaesthetised *mdx* mouse across a range of behaviours—baseline, following vagotomy (increased central respiratory drive) and during exposure to hypercapnic hypoxia (further increase in central respiratory drive)—are shown in Figure 4. Table 1 shows summary data for ventilatory parameters measured directly in anaesthetised *mdx* and *mdx* + NAC mice. There were no significant differences between *mdx* and *mdx* + NAC for respiratory frequency, tidal volume, minute ventilation, and peak inspiratory and expiratory flows across all behaviours. Consistent with observations in conscious mice, NAC treatment had no effect on ventilatory performance in the *mdx* mouse model of DMD.

**TABLE 1:** Respiratory parameters during baseline, following vagotomy and during exposure to hypercapnic hypoxia (HcHx) in *mdx* and *mdx* + NAC mice.

	*mdx* (*n* = 15)	*mdx* + NAC (*n* = 13)	*P‐value*
Baseline			
Respiratory frequency (breaths/min)	191 ± 39	196 ± 36	0.9849
Tidal volume (μL/g)	5.8 ± 1.3	5.1 ± 1.5	0.5162
Minute ventilation (mL/g/min)	1.08 ± 0.19	0.98 ± 0.23	0.5710
Peak inspiratory flow (mL/s)	2.94 ± 0.51	2.82 ± 0.55	0.9309
Peak expiratory flow (mL/s)	4.61 ± 0.65	5.05 ± 0.61	0.2216
Vagotomy			
Respiratory frequency (breaths/min)	48 ± 7	42 ± 7	0.0888
Tidal volume (μL/g)	15.0 ± 6.0	15.3 ± 6.6	0.9989
Minute ventilation (mL/g/min)	0.70 ± 0.25	0.63 ± 0.25	0.8402
Peak inspiratory flow (mL/s)	7.68 ± 1.49	8.57 ± 1.75	0.4169
Peak expiratory flow (mL/s)	6.89 ± 1.32	7.84 ± 1.55	0.2613
HcHx			
Respiratory frequency (breaths/min)	35 ± 8	32 ± 4	0.4617
Tidal volume (μL/g)	21.6 ± 6.6	21.3 ± 8.8	0.9994
Minute ventilation (mL/g/min)	0.75 ± 0.24	0.67 ± 0.25	0.7965
Peak inspiratory flow (mL/s)	9.43 ± 2.27	10.71 ± 1.2	0.1967
Peak expiratory flow (mL/s)	9.38 ± 1.88	10.12 ± 1.41	0.5754

*Note*: Values are expressed as means ± standard deviation (SD) and were statistically compared by repeated measures two‐way ANOVA (or mixed model when occasional data points were missing for technical reasons) with Šidák's multiple comparisons *post hoc* test (Prism 10.2.0). HcHx, hypercapnic hypoxia. Exact *P*‐values are reported for all comparisons.

**FIGURE 5:**
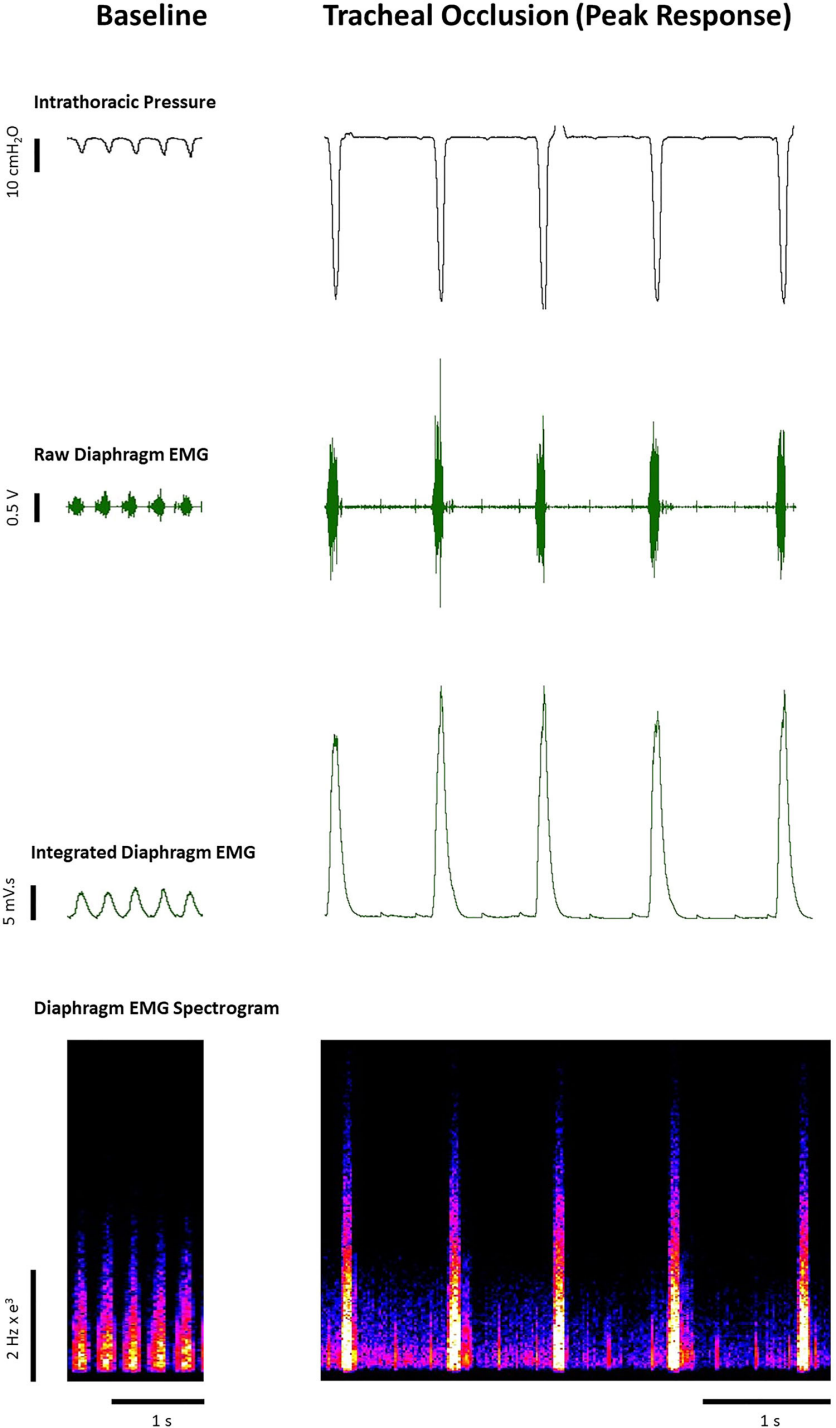
Original traces of respiratory parameters during baseline and tracheal occlusion in an anaesthetised *mdx* mouse. Diaphragm electromyogram activity including raw, integrated and spectrogram traces, together with tracheal airflow and tidal volume in an anaesthetised *mdx* mouse during baseline and sustained tracheal occlusion.

**FIGURE 6:**
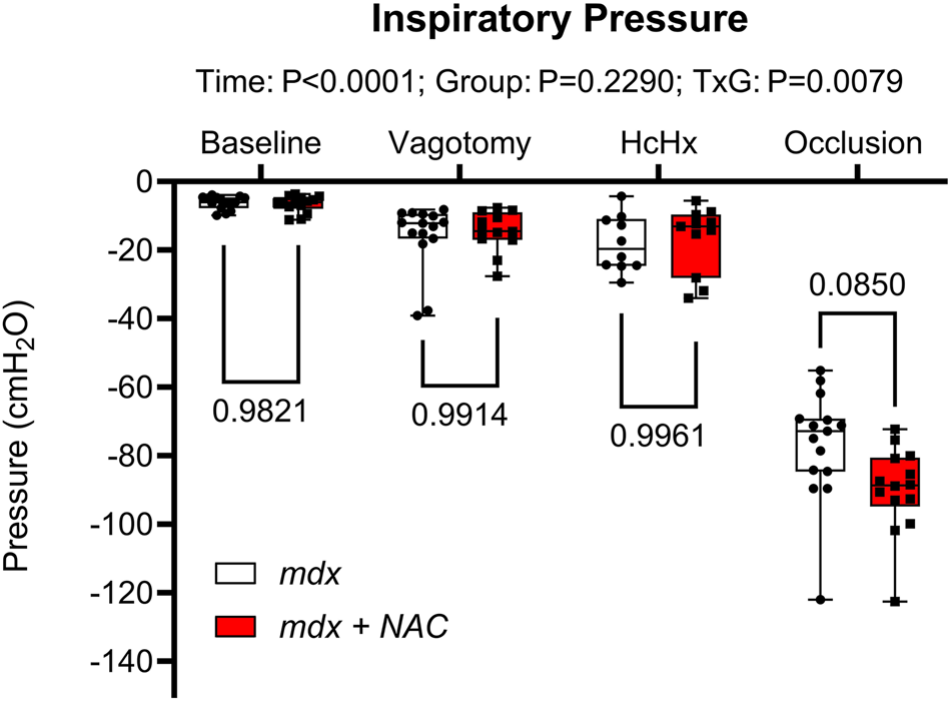
Inspiratory pressure during baseline, following vagotomy, during hypercapnic hypoxia (HcHx) exposure and tracheal occlusion in *mdx* and *mdx* + NAC mice. Values are expressed as box and whisker plots (median, 25−75 percentile and scatter plot) and were statistically compared by repeated measures two‐way ANOVA (or mixed model when occasional data points were missing for technical reasons) with Šidák's multiple comparisons *post hoc* test (Prism 10.2.0). Exact *P*‐values are reported for all comparisons.

Representative original traces of inspiratory pressure and diaphragm EMG activity at baseline and during tracheal occlusion are shown in Figure 5. Table 1 and Figure 6 illustrate the inspiratory pressure and EMG activity of obligatory and accessory muscles of breathing during baseline conditions, following bilateral vagotomy, during hypercapnic hypoxia and during sustained tracheal airway occlusion. There were no significant differences between *mdx* and *mdx* + NAC mice across all behaviours. Chronic NAC treatment had no effect on inspiratory pressure generating capacity in the *mdx* mouse model of DMD.

**FIGURE 7:**
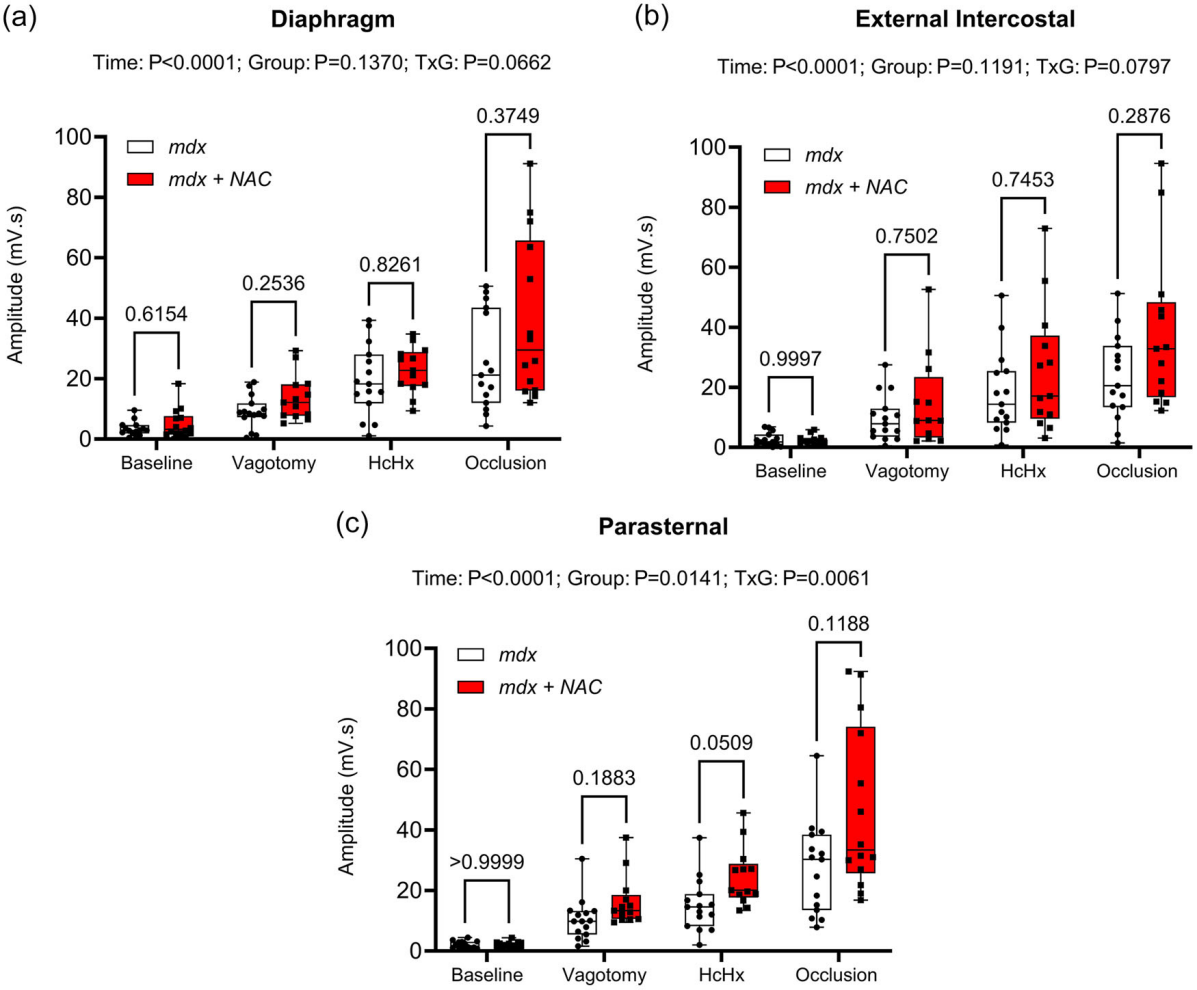
Diaphragm (a), external intercostal (b) and parasternal (c) respiratory EMG activity during baseline, following vagotomy, during hypercapnic hypoxia (HcHx) exposure and during tracheal occlusion in *mdx* and *mdx* + NAC mice. Values are expressed as box and whisker plots (median, 25−75 percentile and scatter plot) and were statistically compared by repeated measures two‐way ANOVA (or mixed model when occasional data points were missing for technical reasons) with Šidák's multiple comparisons *post hoc* test (Prism 10.2.0). Exact *P*‐values are reported for all comparisons.

Figure 7 shows EMG activity of the obligatory muscles of breathing (diaphragm, external intercostal and parasternal intercostal muscles) during baseline, following bilateral vagotomy, during hypercapnic hypoxia exposure and during sustained tracheal airway occlusion. Chronic NAC treatment had no effect on the amplitude of obligatory respiratory muscle EMG activity of the *mdx* mouse model of DMD.

**FIGURE 8:**
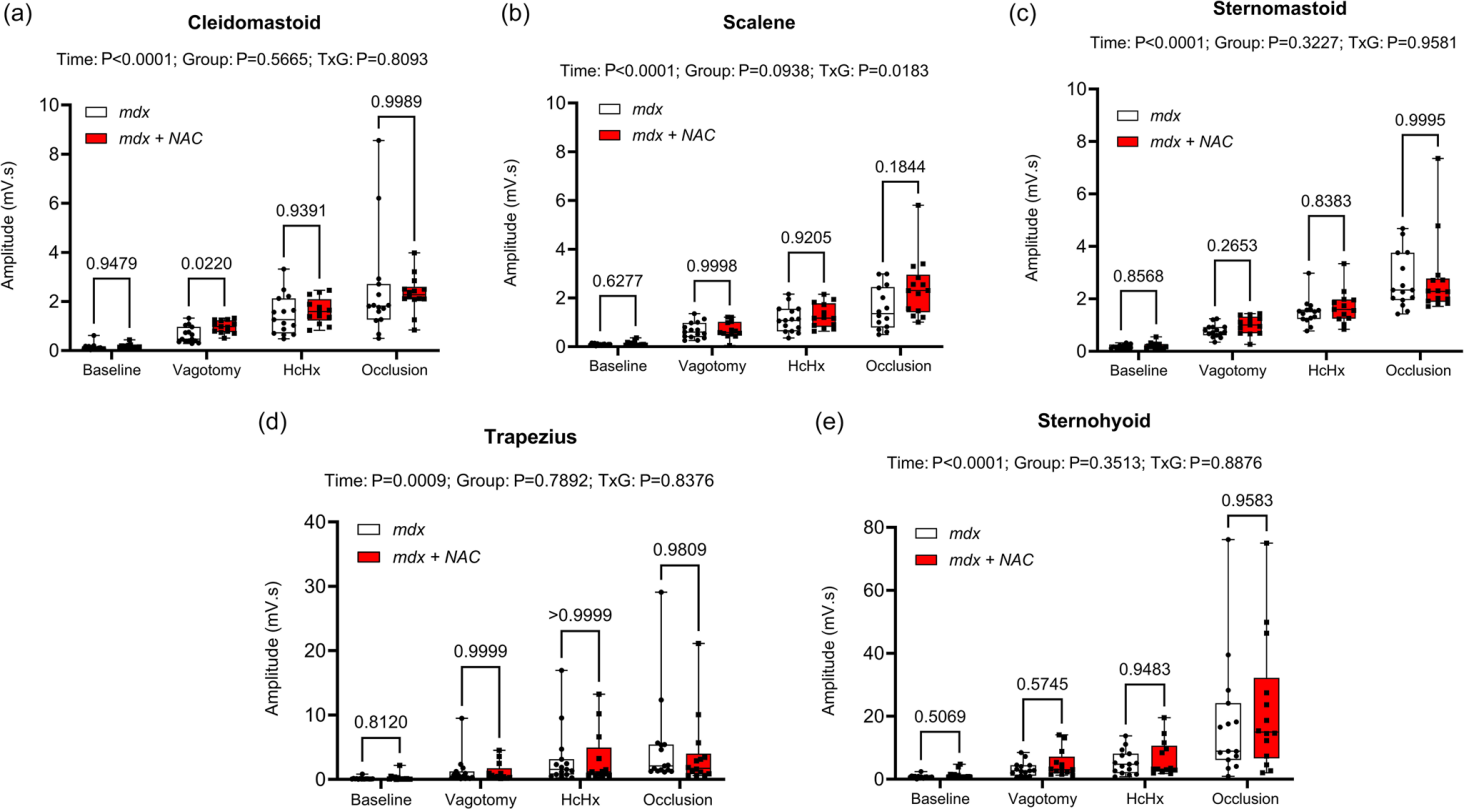
Cleidomastoid (a), scalene (b), sternomastoid (c), trapezius (d) and sternohyoid (e) respiratory EMG activity during baseline, following vagotomy, during hypercapnic hypoxia (HcHx) exposure and during tracheal airway occlusion in *mdx* and *mdx* + NAC mice. Values are expressed as box and whisker plots (median, 25−75 percentile and scatter plot) and were statistically compared by repeated measures two‐way ANOVA (or mixed model when occasional data points were missing for technical reasons) with Šidák's multiple comparisons *post hoc* test (Prism 10.2.0). Exact *P*‐values are reported for all comparisons.

Figure 8 shows summary data for EMG activity of the accessory muscles of breathing (cleidomastoid, scalene, sternomastoid, trapezius and sternohyoid) during baseline, following bilateral vagotomy, during hypercapnic hypoxia exposure and during sustained tracheal airway occlusion. Chronic NAC treatment had no effect on EMG activity of the accessory muscles of breathing across a range of behaviours in the *mdx* mouse model of DMD.

### 3.5 Inflammatory cell infiltration and collagen deposition in diaphragm muscle

**FIGURE 9:**
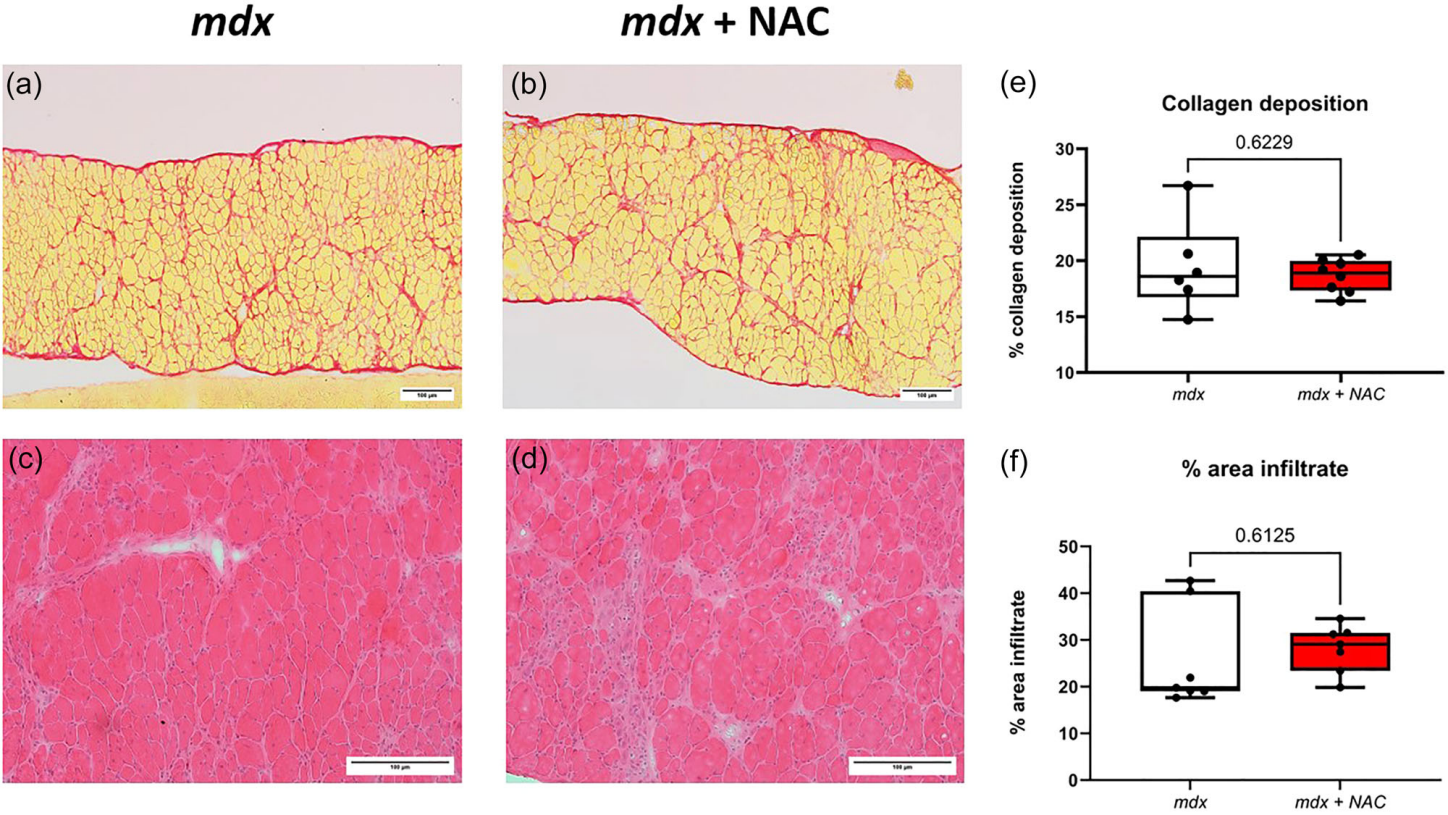
Collagen deposition and immune cell infiltration in diaphragm muscle of *mdx* and *mdx* + NAC mice. Representative histological images of transverse sections of diaphragm muscle stained with Sirius red (a, b), and haematoxylin and eosin (c, d) in *mdx* and *mdx* + NAC mice. Group data showing the relative area of collagen deposition (e), and relative area of infiltration of inflammatory cells (f). Values are expressed as box and whisker plots (median, 25−75 percentile and scatter plot) and were statistically compared using unpaired two‐tailed *t* tests. Exact *P*‐values are reported for comparisons between groups.

Figure 9 shows representative histological images for diaphragm muscle from *mdx* and *mdx* + NAC groups. Collagen deposition in *mdx* and *mdx* + NAC diaphragms is equivalent. Similarly, percentage area of immune cell infiltration was equivalent between *mdx* and *mdx* + NAC. Chronic NAC treatment had no effect on immune cell infiltration or collagen deposition (fibrosis) in the *mdx* mouse model of DMD.

### 3.6 Diaphragm muscle mRNA expression

**FIGURE 10:**
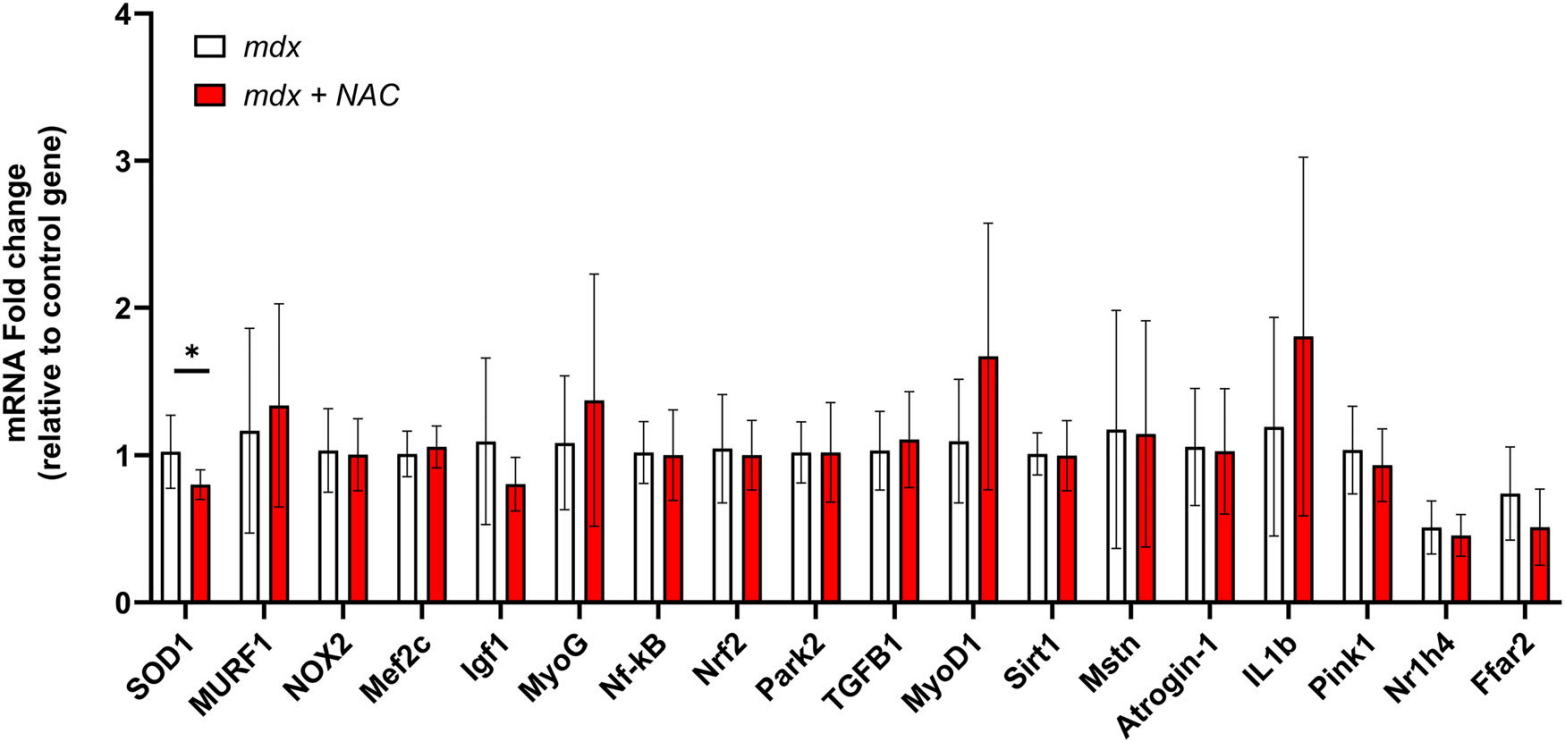
Diaphragm muscle mRNA expression. Group data for mRNA expression of *SOD1*, *MURF1*, *NOX2*, *Mef2c*, *Igf1*, *MyoG*, *Nf‐kB*, *Nrf2*, *Park2*, *TGFB1*, *MyoD1*, *Sirt1*, *Mstn*, *Atrogin‐1*, *IL1b*, *Pink1*, *Nr1h4*, *Ffar2* in diaphragm muscle from *mdx* (*n* = 10) and *mdx* + NAC (*n* = 10) groups. Data are shown as means ± SD and were statistically compared using two‐tailed, unpaired Mann–Whitney tests. *Ffar2*, free fatty acid receptor 2; *IGF*, insulin‐like growth factor; *IL1B*, interleukin 1 beta; *MEF2C*, myocyte enhancer factor 2C; *Mstn*, myostatin; *Murf‐1*, muscle RING‐finger protein‐1; *MyoD*, myogenic differentiation; *MyoG*, myogenin; *NFκB*, nuclear factor kappa‐B; *NOX*, NADPH oxidase; *Nr1h4*, nuclear receptor subfamily 1 group H member 4; *Nrf2*, nuclear factor erythroid 2‐related factor 2; *PARK‐2*, parkin RBR E3 ubiquitin protein ligase; *Pink1*, phosphatase and tensin homolog (PTEN)‐induced kinase 1; *Sirt1*, sirtuin‐1; *SOD1*, superoxide dismutase 1; *TGFB‐1*, transforming growth factor‐B. *represents *P* = 0.022.

Figure 10 shows mRNA expression of genes related to antioxidant capacity, autophagy, mitophagy, muscle repair and inflammation from diaphragm samples of *mdx* and *mdx* + NAC mice. There was a significant decrease in *SOD1* in the *mdx* + NAC group compared to the *mdx* group. There were no significant differences in mRNA fold change for any other genes tested.

## 4 DISCUSSION

The major findings of this study are: (i) chronic NAC treatment did not improve *mdx* diaphragm functional capacity; (ii) chronic NAC treatment did not reduce collagen deposition (fibrosis) and immune cell infiltration in *mdx* diaphragm; (iii) chronic NAC had no effect on ventilation and ventilatory responsiveness in *mdx* mice; (iv) chronic NAC did not improve respiratory muscle EMG activity in *mdx* mice across a range of behaviours from basal to peak activity.

Previous studies have shown that diaphragm muscle force is reduced in the *mdx* model when compared to wild‐type mice, equating to 40%–50% loss of peak force by 4 months of age (Burns et al., 2018, Burns, Drummond et al., 2019; O'Halloran et al., 2023). The present study demonstrates that chronic NAC treatment (1% NAC for 3 months) does not rescue diaphragm muscle force‐generating capacity, such that *mdx* + NAC treated mice generated forces equivalent to *mdx*, which are considerably lower than wild‐type values (Burns et al., 2018, Burns, Drummond et al., 2019; O'Halloran et al., 2023). NAC was ineffective in improving diaphragm muscle force across a range of stimulus intensities, demonstrating a lack of effect on muscle force relevant to basal, sub‐maximal and maximal behaviours, that is, resting breathing, peak ventilatory capacity and peak respiratory system performance.

Structural remodelling is evident in dystrophic muscle, including increased central nucleation (indicative of fibre regeneration following damage), immune cell infiltration (inflammatory response) and collagen content (fibrosis) (Burns, Drummond et al., 2019; O'Halloran et al., 2023). Our study revealed that chronic NAC treatment did not ameliorate canonical dystropathology in the *mdx* mouse over the period of 1– 4 months of age.

We investigated the effects of NAC treatment on ventilation and ventilatory responsiveness to hypercapnic hypoxia in conscious and anaesthetised mice, and inspiratory pressure and respiratory (obligatory and accessory) EMG activity in anaesthetised mice. These assessments confirmed the capacity for *mdx* mice to maintain basal breathing similar to wild‐type mice and enhance ventilation in response to ventilatory challenges as previously described (Burns, Murphy et al., 2019); NAC treatment had no effect on breathing. Respiratory airflow and volumes were measured in anaesthetised mice during baseline, following bilateral vagotomy and with superimposed chemo‐activation to maximally activate ventilation. Tidal volume, peak inspiratory and expiratory flow rates and minute ventilation were equivalent between groups. Considering respiratory parameters are generally well protected in *mdx* mice at 4 months of age (O'Halloran et al., 2023), these data are unsurprising, yet nevertheless demonstrate no adverse effects of NAC treatment on breathing in early dystrophic disease.

We have previously described reduced respiratory EMG activity during maximum activation in anaesthetised *mdx* mice compared with age‐matched wild‐type mice (Burns, Drummond et al., 2019; O'Halloran et al., 2023). Decreased respiratory EMG activity evident as reduced motor unit potential amplitudes most likely reflects impaired neuromuscular transmission due to neuromuscular junction dysfunction in muscular dystrophy and/or loss of large motor units (Carlson & Roshek, 2001; Personius & Sawyer, 2006). The present study demonstrates that chronic NAC treatment does not rescue impaired respiratory EMG activity, further illustrating no apparent benefit of chronic administration of NAC during the progression from early to established muscular dystrophy.

Our examination of diaphragm mRNA expression indicated a decrease in the expression levels of *SOD1* in *mdx* + NAC presumably related to the antioxidant effects of NAC. However, transcriptional responses in *mdx* diaphragm, characterised by increased pro‐oxidant, pro‐inflammatory and pro‐fibrotic factors, in addition to those associated with increased muscle regeneration (Burns, Drummond et al., 2019) were unaffected by chronic NAC treatment.

A comparison of the present study to our previous report (Burns, Drummond et al., 2019) reveals that short‐term beneficial effects of NAC on diaphragm function (principally related to positive inotropic effects on healthy fibres) is not maintained over a longer time course of disease, albeit that measures following short‐term NAC administration were not performed in the present study to confirm our previous finding. A significant increase in dystropathology of the *mdx* diaphragm manifests in the period from 1 to 4 months of age, including the emergence of increased fibrosis (O'Halloran et al., 2023). It is evident that NAC treatment alone is inadequate to ameliorate the pathophysiological changes that ensue due to injury and inflammation in the principal muscle of breathing. We acknowledge that other studies have employed higher concentrations of NAC, and such studies are required to fully comprehend the potential efficacy of NAC treatment alone for DMD, although chronic treatment with high concentrations of NAC may have side effects (Head, 2017; Pinniger et al., 2017). Sex differences in the efficacy of NAC are reported (Bushana et al., 2023; Goenaga et al., 2020) with potentially greater efficacy in males. All mice in our study were male given that DMD is X‐linked.

The gold standard treatment for DMD is the use of glucocorticoids such as prednisone or deflazacort, which aim to slow disease progression by anti‐inflammatory action (Gloss et al., 2016). Their benefits are well known, but so too are their side effects. Chronic use of steroids is associated with deleterious effects including muscle atrophy (Quattrocelli et al., 2017). Various strategies are undertaken to offset these effects in pre‐clinical trials with a view to translation to human DMD. For example, it has been successfully shown that weekly treatment rather than daily treatment with prednisone is effective and can offset the negative side effects of chronic use of steroidal treatment (Quattrocelli et al., 2017). Quattrocelli et al. (2017) compared weekly versus daily prednisone and deflazacort over a 4‐week period. Weekly treatment with glucocorticoids showed an increase in functional measures including grip strength, run to exhaustion and tetanic force in the tibialis anterior (Quattrocelli et al., 2017). Furthermore, weekly treatment increased gastrocnemius and diaphragm muscle cross‐sectional areas, whereas these measures were decreased following chronic daily treatment (Quattrocelli et al., 2017). We acknowledge that promising emerging therapies will likely be administered as adjunctive therapies in human DMD. It would be interesting to determine if co‐administration of NAC and prednisone exerted beneficial effects on respiratory muscle over prednisone alone.

Notwithstanding the observations of the present study, which may suggest limited application for NAC in the treatment of DMD, we conclude that further studies are warranted to fully investigate the potential beneficial effects of NAC as an adjunctive treatment for muscular dystrophy.

## AUTHOR CONTRIBUTIONS

Michael N. Maxwell: administration of NAC; acquisition of whole‐body plethysmography data; acquisition of diaphragm histology data; data and statistical analysis and interpretation of data; preparation of figures and drafting of the manuscript. Anthony L. Marullo: administration of NAC; acquisition of force data; data and statistical analysis; preparation of figures; drafting of the manuscript. Ben T. Murphy: administration of NAC; critical review of the manuscript.  Esther Valverde‐Pérez: acquisition of gene expression data; data and statistical analysis and interpretation of the data. Aoife D. Slyne: administration of NAC; acquisition of tissue and growth data; critical review of the manuscript. Ken D. O'Halloran: experimental design; acquisition of EMG and pressure data; data and statistical analysis and interpretation of data; preparation of figures and drafting and critical review of the manuscript. Authors have approved the final version of the manuscript and agree to be accountable for all aspects of the work in ensuring that questions related to the accuracy or integrity of any part of the work are appropriately investigated and resolved. All persons designated as authors qualify for authorship, and all those who qualify for authorship are listed.

## CONFLICT OF INTEREST

None.

## Data Availability

Most data are provided in the manuscript as individual data points. All data are available upon reasonable request.
